# Evaluation of intravoxel incoherent motion fitting methods in low‐perfused tissue

**DOI:** 10.1002/jmri.25411

**Published:** 2016-08-22

**Authors:** Emma M. Meeus, Jan Novak, Stephanie B. Withey, Niloufar Zarinabad, Hamid Dehghani, Andrew C. Peet

**Affiliations:** ^1^ Physical Sciences of Imaging in Biomedical Sciences (PSIBS), Doctoral Training CentreUniversity of BirminghamUnited Kingdom; ^2^ Institute of Cancer and Genomic SciencesUniversity of BirminghamUnited Kingdom; ^3^ Department of OncologyBirmingham Children's HospitalBirminghamUnited Kingdom; ^4^ RRPPSUniversity Hospitals Birmingham NHS Foundation TrustBirminghamUnited Kingdom; ^5^ School of Computer ScienceUniversity of BirminghamUnited Kingdom

**Keywords:** diffusion weighted MRI, intravoxel incoherent motion, IVIM, brain tumors, perfusion

## Abstract

**Purpose:**

To investigate the robustness of constrained and simultaneous intravoxel incoherent motion (IVIM) fitting methods and the estimated IVIM parameters (D, D* and *f*) for applications in brain and low‐perfused tissues.

**Materials and Methods:**

Model data simulations relevant to brain and low‐perfused tumor tissues were computed to assess the accuracy, relative bias, and reproducibility (CV%) of the fitting methods in estimating the IVIM parameters. The simulations were performed at a series of signal‐to‐noise ratio (SNR) levels to assess the influence of noise on the fitting.

**Results:**

The estimated IVIM parameters from model simulations were found significantly different (*P* < 0.05) using simultaneous and constrained fitting methods at low SNR. Higher accuracy and reproducibility were achieved with the constrained fitting method. Using this method, the mean error (%) for the estimated IVIM parameters at a clinically relevant SNR = 40 were *D* 0.35, D* 41.0 and *f* 4.55 for the tumor model and *D* 1.87, D* 2.48, and *f* 7.49 for the gray matter model. The most robust parameters were the IVIM‐*D* and IVIM‐*f*. The IVIM‐D* was increasingly overestimated at low perfusion.

**Conclusion:**

A constrained IVIM fitting method provides more accurate and reproducible IVIM parameters in low‐perfused tissue compared with simultaneous fitting.

**Level of Evidence**: 3

J. MAGN. RESON. IMAGING 2017;45:1325–1334

Diffusion‐ and perfusion‐weighted MRI methods are becoming more prevalent in clinical practice due to their ability to provide information about tissue microstructure such as cellularity and vascularity, respectively.^1^, ^2^ Diffusion‐weighted imaging (DWI) has the advantage of being noninvasive with no requirement for an intravenous contrast agent, whereas contrast is needed for perfusion methods such as dynamic susceptibility contrast (DSC) imaging. In brain tumors, DWI has been shown to improve tissue characterization,^3^ monitor treatment response,^4^ differentiate posttherapeutic changes from active tumor residuals,^5^ and aid in tumor staging.^6^ While DWI can provide an insight into many microstructural features alone, using it in combination with perfusion‐derived parameters could result in a more complete investigation.

Recently, the intravoxel incoherent motion (IVIM) model has gained more interest with its derivation of diffusion and perfusion related parameters from DWI sequences.^7^ This is achieved by using different magnitudes of diffusion weighting.^8^, ^9^ The overall IVIM signal decay can be modelled as:

(1) $$\frac{S\left(b\right)}{S\left(0\right)}=f\cdot {\text{exp}}^{-bD*}+\left(1-f\right)\cdot {\text{exp}}^{-bD}$$

where *S*(*b*)/*S*(0) is the signal intensity at a certain *b*‐value normalized by the signal intensity at *b* = 0, *D* is the tissue diffusion coefficient, D* is the pseudo‐diffusion coefficient, and *f* is the perfusion fraction describing the fraction of signal arising from the vascular network.

Links between the IVIM perfusion parameters f, D*, and fD* have been established to classical perfusion parameters cerebral blood volume (CBV), mean transit time (MTT), and cerebral blood flow (CBF), respectively.^10^ However, while IVIM‐*f* was found to correlate well with DSC‐CBV in healthy adult gray matter^11^ and in low‐ and high‐grade gliomas,^12^ the IVIM‐D* and IVIM‐fD* have produced more contradictory results.^11^, ^13^ These studies suggest that the bi‐exponential behavior in the brain might not be sufficiently pronounced for the robust computation of IVIM‐D*.

The reliable derivation of the IVIM parameters depends on the chosen DWI protocol (e.g. number of averages and *b*‐values)^14^, ^15^ and the subsequent postprocessing of the image data.^16^ Barbieri et al^16^ have previously studied the fitting of the IVIM signal data in detail, where six fitting algorithms were compared for upper abdominal organs. Variability was observed between the algorithms in all abdominal regions, which included the simultaneous (three‐parameter) and constrained (one‐parameter) fitting methods also used in this study. However, the study did not include the fitting method (two‐parameter) more commonly used in previous IVIM brain studies.^11^, ^12^, ^13^ The application of the IVIM model in the brain differs from the abdomen in terms of the degree of perfusion or bi‐exponential behavior of the observed signal. The perfusion in the brain is generally lower when compared with the abdomen and, therefore, poses a greater challenge for the bi‐exponential IVIM model. Low‐perfused tissue has been observed in regions of acute ischemic stroke,^13^ traumatic brain injury,^17^ and both adult and pediatric low‐grade brain tumors.^12^, ^18^ The assessment of low‐perfused tissue with the IVIM model could provide a noninvasive alternative to the more commonly used DSC‐MRI. The purpose of this study was to assess and compare the constrained and simultaneous IVIM fitting methods in low‐perfused tissue.

## Materials and Methods

### Data Simulations

Model data simulations were performed to investigate the effects of the fitting algorithms on the accuracy and reproducibility of the estimated IVIM parameters. All simulations and data analysis were implemented using in‐house Python software (Anaconda, Continuum Analytics, v. 2.7.10) with the LmFit library.^19^ The model data signal values were generated with Eq. [(1)] using the same *b*‐value distribution as used for the patient imaging (*b* = 0, 20, 40, 80, 110, 140, 170, 200, 300, 500, 1000 s/mm^2^). The IVIM parameters for the gray matter (GM) and tumor model were obtained using the average values of the corresponding regions from the patient cohort. The following IVIM values were used for the GM model: *D* = 8.32 × 10^−4^ mm^2^/s, D* = 2.68 × 10^−2^ mm^2^/s, *f* = 0.115 and tumor model: *D* = 1.63 × 10^−3^ mm^2^/s, D* = 7.23 × 10^−3^ mm^2^/s, and *f* = 0.0953.

**Figure 1:**
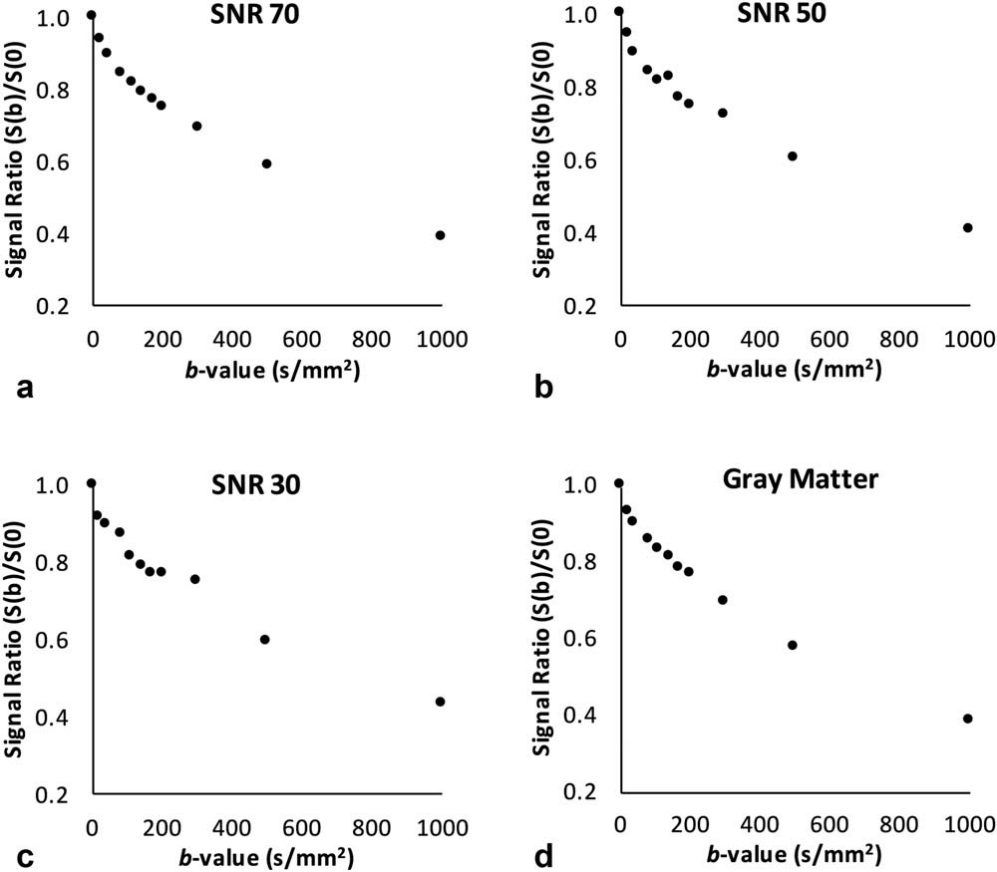
An example of simulated bi‐exponential gray matter signal created for the different SNR levels of 70, 50, and 30 (**a–c**) and experimental diffusion signal (**d**) derived from a healthy gray matter region.

Random white Gaussian noise was introduced to the model data to mimic SNR levels of 20 to 70 based on (Eq. [(2)]):

(2) $$\text{SNR}=\;\frac{\mu }{\sigma }$$

where *μ* is the maximum signal and *σ* is the standard deviation of the noise. The noise was created using a Gaussian function, which returned a symmetric Gaussian filter with appropriate values of μ and *σ* for the chosen SNR level. Random values from the filter were added to the simulated data to create sets of signal data with random noise. An example of the generated gray matter signal data at different SNR levels is shown in Figure 1 together with an experimental signal derived from a healthy gray matter region. SNR levels were selected based on previous publications^20^, ^21^ and the patient cohort in our study to cover appropriate noise levels observed in diffusion‐weighted imaging. The SNR values were determined with the standard NEMA method^22^ from the DWI protocol using dynamic imaging. The SNR values at *b* = 1000 s/mm^2^ were found to be in the range of 35–53. The data simulations were performed using 1000 random data iterations for each model and SNR level. Data iterations with ill‐conditioned Jacobian matrix were defined as outliers.

### MRI

All MR imaging was performed on a Philips Achieva 3 Tesla (T) TX (Philips Healthcare, Best, the Netherlands) MRI scanner with a 32‐multichannel receive head coil at Birmingham Children's Hospital, United Kingdom. Seven different brain tumor patients (age 1.6 to 10.2 years; mean age, 4.6 years) were scanned. The tumor types included: hypothalamic/chiasmatic pilocytic astrocytoma,^1^ glioneuronal tumor,^1^ optic pathway glioma,^4^ and hypothalamic glioma.^1^ Informed parental consent was obtained for all subjects and the East Midlands – Derby Research Ethics Committee (REC 04/MRE04/41) approved the study operating under the rules of Declaration of Helsinki 1975 (and as revised in 1983). The DW‐MRI protocol used a sensitivity‐encoded (SENSE) approach with single‐shot, spin echo planar imaging sequence. For each subject 11 exponentially spaced *b*‐values were acquired in three orthogonal directions with TR/TE = 4000/91 ms, field of view (FOV) 240 × 240 mm^2^, matrix size 96 × 96, slice thickness 3.5 mm with 30 contiguous axial slices and in‐plane resolution of 2.5 × 2.5 mm^2^. The total scan duration was 2.12 min.

### Data Analysis

**Figure 2:**
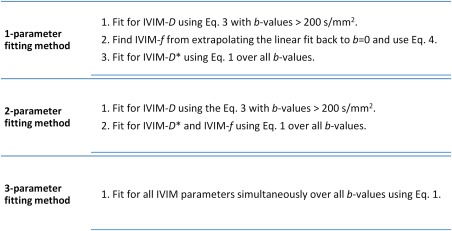
Bi‐exponential IVIM fitting methods used in this study using the Levenberg‐Marquardt algorithm with least‐squares minimization.

Nonlinear least squares minimization was performed with the Levenberg‐Marquardt algorithm for the IVIM fitting of signal data. The fitting was performed on a voxel‐by‐voxel basis using the bi‐exponential methods outlined in Figure 2, which varied from a nonconstrained simultaneous fitting to a more constrained step‐wise fitting with fixed IVIM parameters.

For the one‐ and two‐parameter fitting, the model assumed that the perfusion effects were negligible at high *b*‐values ( > 200 s/mm^2^)^23^; therefore, the mono‐exponential relationship was be used to define IVIM‐*D* (Eq. [(3)]).

(3) $$\frac{S\left(b\right)}{S\left(0\right)}={\text{exp}}^{-bD}$$

Additionally, the one‐parameter method used the linear fit from Eq. [(3)] to derive the IVIM‐*f* value by extrapolating the fit to the *y*‐intercept, *S*(int). When no vascular component is present, *S*(int) = *S*(0). The IVIM‐*f* parameter can be defined as:

(4) $$f=\frac{S\left(0\right)-S\left(\text{int}\right)}{S\left(0\right)}$$

The reproducibility was computed in terms of coefficient of variation (CV) for each IVIM parameter at each SNR and determined as the percentage of the ratio of standard deviation of the mean parameter to the mean parameter for a set of model data iterations. Similarly, the Bland‐Altman analysis of relative bias was computed for each parameter as a percentage of the difference between the ground truth IVIM parameters and the estimated IVIM parameters with limits of agreement determined from the standard deviation of the mean difference (95% confidence intervals).

### Statistical Analysis

The statistical analysis was performed in SPSS Statistics (IBM, Chicago, IL, v.22). Analysis of variance (ANOVA) was performed for the repeated IVIM model data simulations and the estimated parameter values derived using the different fitting methods. This was to test if estimated parameters differed significantly (*P* < 0.05) between the fitting algorithms. The Tukey post hoc analysis was used to define which algorithms differed significantly (*P* < 0.05). The model data were further analyzed using confidence intervals and the resulting 2D error norm plots computed with the LmFit library.^19^ The method used an F‐test to compare the null model (best fit) with an alternate model where one parameter value was fixed.

## Results

### Model Data Simulations

**Figure 3:**
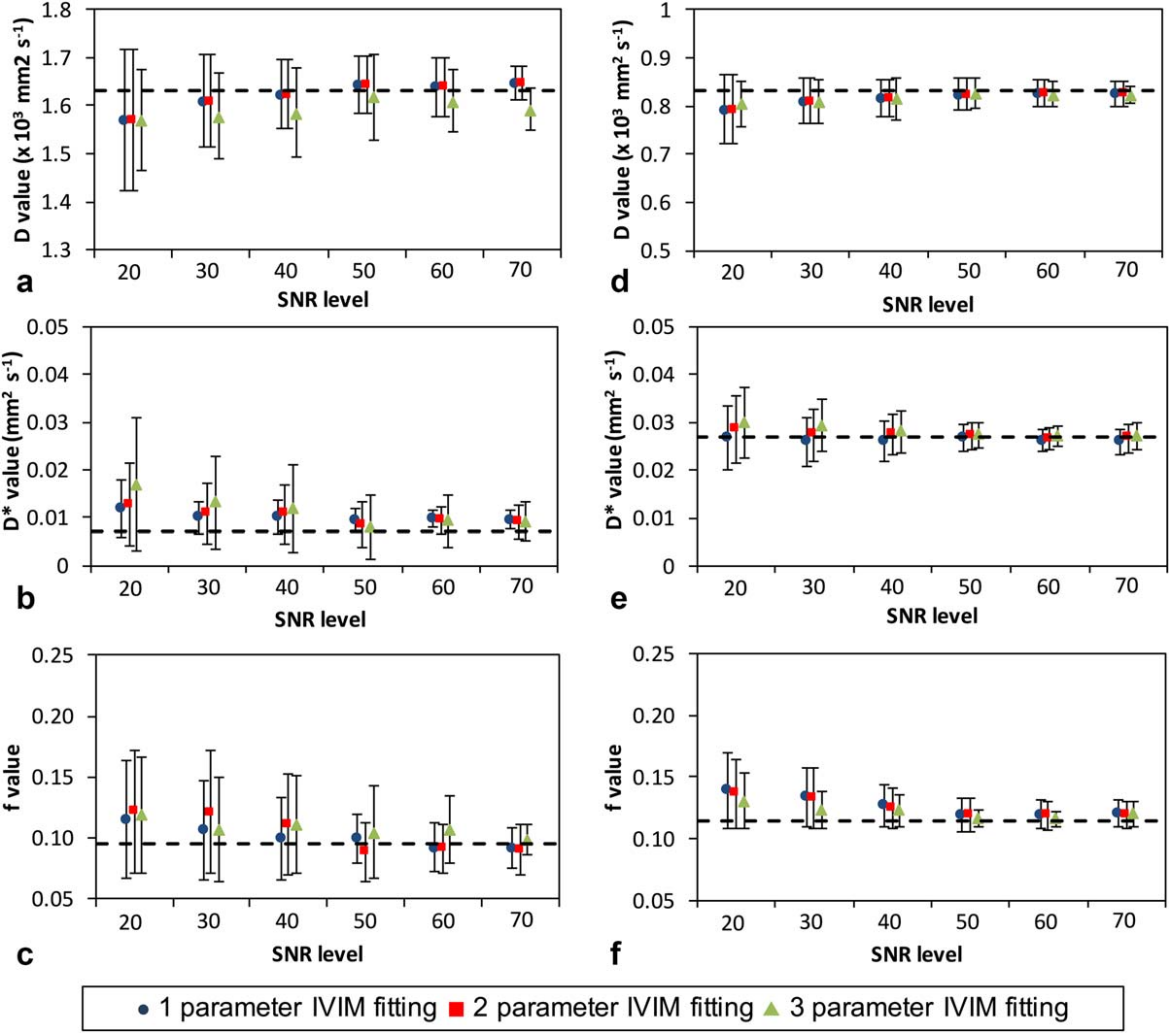
The accuracy results for the estimated IVIM parameters from the data simulations using the different fitting methods with tumor (**a–c**) and gray matter model (**d–f**). The true IVIM values are shown as dashed lines and error bars represent the standard deviation of the estimation.

The tumor and gray matter simulation results for the fitting methods and the estimation of IVIM‐*D*, IVIM‐D*, and IVIM‐*f* parameters are shown in Figure 3 and Tables 1 and 2. The absolute accuracy was determined using the true IVIM values and the measured mean value obtained from fitting of the simulated data (Fig. 3). The estimation of IVIM‐*D* (Fig. 3a,d) was robust for both the constrained and simultaneous fitting for tumor and gray matter model. At lower SNR levels the mean IVIM‐*D* decreased, which lead to an increase of both IVIM‐*f* and IVIM‐D* mean values (Fig. 3b,c,e,f). While increasing variance was observed for all the IVIM parameters at decreasing SNR levels, the variance was comparatively reduced for the IVIM‐*D* and IVIM‐*f*.

**Table 1:** Reproducibility (Coefficient of variation, CV%) and Outlier Results for the Model Simulation*

	Tumor IVIM‐*D*		Tumor IVIM‐*D* *			Tumor IVIM‐*f*			Tumor outliers %			GM IVIM‐*D*		GM IVIM‐*D* *			GM IVIM‐*f*			GM Outliers %		
SNR	1‐2	3	1	2	3	1	2	3	1	2	3	1‐2	3	1	2	3	1	2	3	1	2	3
70	4.29	5.51	35.0	64.4	104	32.8	39.2	44.1	11.3	21.7	30.1	6.34	6.26	20.2	16.9	16.1	11.9	10.7	8.48	9.1	10.2	19.6
60	7.42	7.93	42.6	66.6	118	40.3	44.8	54.5	12.9	23.6	49.2	6.51	7.25	20.8	22.2	21.1	18.4	17.8	17.6	11.5	12.6	22.4
50	7.53	11.0	52.1	75.9	142	48.8	52.9	70.3	20.5	29.9	54.9	7.78	7.73	21.6	28.5	31.9	19.2	19.6	20.7	18.5	24.5	35.7
40	8.67	11.4	58.3	101	150	50.9	54.8	73.3	26.7	42.0	65.6	9.54	10.4	21.7	31.0	36.3	22.3	22.8	23.7	23.3	28.7	43.7
30	11.8	11.7	68.5	113	152	60.2	74.8	80.3	28.4	49.9	70.1	11.7	11.2	32.3	40.2	48.7	26.5	26.0	28.7	27.7	31.5	54.6
20	18.8	13.3	69.9	118	164	77.6	82.4	80.5	34.2	54.7	73.0	18.2	11.5	39.7	49.8	55.7	35.8	36.8	34.1	31.8	37.3	55.7

*The numbering 1–3 refer to the corresponding parameter fitting methods.

A significant difference between the algorithms was observed in the tumor model for the estimation of mean IVIM‐*D* (SNR 30–70; *P* = 0.001–0.005), IVIM‐D* (SNR 20–30; *P* = 0.001) and IVIM‐*f* (SNR 30–60; *P* = 0.001–0.008) values. This was shown by the post hoc test to be due to the difference between the constrained fitting methods and the three‐parameter fitting. Better accuracy was achieved with the constrained fitting methods (Fig. 3a–c). The constrained fitting methods (one‐ and two‐parameter) produced different values of IVIM‐*f* at SNR 30–40. In the gray matter model, significant differences were observed for the estimation of the mean IVIM‐D* (SNR 20–30; *P* = 0.001) and IVIM‐*f* (SNR 20–30; *P* = 0.001–0.031) values. Similar to the tumor model, the difference arose in both the constrained and simultaneous fitting methods.

The IVIM parameter reproducibility (coefficient of variation, CV%) and the number of outliers from the fitting methods are reported in Table 1 and the relative bias (%) with limits of agreement in Table 2. The gray matter model resulted in higher reproducibility and a smaller number of outliers compared with the tumor model. The three‐parameter fitting was the most reproducible for the gray matter model, but generated the largest number of outliers. The relative bias between the ground truth IVIM parameters and the estimated IVIM parameters in the gray matter model showed very similar behavior between the fitting methods as shown in Figure 3. For the low‐perfused tumor model, the more constrained fitting methods increased the reproducibility with one‐parameter fitting being the most reproducible overall with the lowest number of outliers. The relative bias for the tumor model indicated similar accuracy between the fitting methods. However, the variance for the estimation of IVIM‐D* was increased using the three‐parameter fitting.

**Table 2:** Bland‐Altman Results of Bias (%) and Limits of Agreement (95% CI) for the Model Simulations*

	Tumor IVIM‐*D*		Tumor IVIM‐*D* *			Tumor IVIM‐*f*			GM IVIM‐*D*		GM IVIM‐*D* *			GM IVIM‐*f*		
SNR	1 & 2	3	1	2	3	1	2	3	1 & 2	3	1	2	3	1	2	3
**Bias(%) Bland‐Altman results of bias (%)**																
70	0.41	−3.12	13.40	13.60	5.33	−10.90	−10.40	1.38	−1.03	−1.28	−3.97	0.90	−0.42	0.41	0.90	0.61
60	0.11	−3.09	13.40	13.90	3.35	−10.00	−10.10	4.28	−0.92	−1.41	−4.73	−1.54	−0.32	0.43	1.21	0.38
50	0.51	−3.72	13.30	10.90	0.46	6.45	−11.60	3.45	−1.33	−0.76	−6.69	−2.26	0.88	1.20	0.50	0.82
40	−1.72	−4.24	13.50	15.00	11.00	3.20	4.29	5.14	−2.50	−2.83	−7.54	1.36	1.01	3.98	0.80	2.89
30	−3.17	−4.32	11.00	24.60	16.80	6.43	11.70	3.31	−3.67	−3.60	−9.10	3.91	4.72	6.35	2.46	2.90
20	−8.41	−8.19	16.30	26.50	21.90	11.50	12.30	11.20	−7.22	−4.15	−9.51	12.20	5.60	8.09	6.08	4.74
**Bland‐Altman limits of agreement (%)**																
70	−14.1, 14.9	−5.88, 6.07	−16.4, 63.2	−35.8, 63.0	−53.6, 64.3	−64.5, 24.7	−55.8, 35.1	−28.3, 31.1	−16.6, 14.6	−16.4, 13.9	−61.4, 51.9	−59.0, 60.8	−58.4, 57.6	−24.1, 26.4	−22.0, 23.6	−27.9, 29.1
60	−18.2, 19.2	−21.9, 19.7	−13.9, 60.6	−35.4, 63.1	−57.9, 64.6	−65.3, 25.4	−56.7, 36.7	−50.1, 37.4	−19.4, 17.5	−25.0, 22.2	−68.1, 60.2	−58.8, 55.8	−59.2, 59.3	−25.7, 26.5	−26.5, 26.5	−30.9, 31.6
50	−20.9, 21.1	−21.7, 20.3	−13.4, 50.0	−69.5, 55.2	−60.5, 61.4	−57.4, 14.4	−91.5, 69.2	−57.5, 44.4	−25.1, 22.4	−10.7, 9.21	−65.2, 51.8	−63.6, 59.0	−61.5, 90.5	−32.5, 33.3	−28.6, 29.6	−28.9, 30.5
40	−26.3, 23.9	−24.4, 22.0	−37.5, 55.6	−63.4, 93.3	−79.3, 101	−78.6, 56.3	−76.9, 77.5	−51.8, 62.0	−27.8, 22.8	−32.7, 27.0	−98.3, 73.3	−45.9, 43.2	−92.5, 90.5	−39.4, 47.4	−29.8, 31.6	−45.2, 51.0
30	−22.7, 39.2	−24.6, 24.0	−39.4, 81.4	−64.3, 93.5	−79.4, 101	−92.7, 79.8	−84.2, 91.6	−51.0, 67.6	−36.7, 29.4	−33.6, 26.3	−117, 78.4	−110, 103	−99.5, 103	−39.9, 52.6	−50.6, 55.5	−50.7, 56.5
20	−44.9, 38.0	−26.1, 26.5	−51.0, 99.1	−71.9, 111	−84.4, 98.2	−97.4, 93.0	−84.6, 93.2	−69.2, 73.3	−61.7, 47.3	−33.7, 25.4	−148, 88.9	−134, 110	−110, 104	−52.1, 68.3	−57.6, 69.9	−54.1, 63.6

*The numbering 1–3 refer to the corresponding parameter fitting methods.

Overall, the one‐parameter fitting method estimated the most accurate and precise IVIM‐*D*, IVIM‐D*, and IVIM‐*f* values. At SNR of 40, the mean error % / reproducibility (CV%) for IVIM‐*D*, IVIM‐D*, and IVIM‐*f* were 0.35/8.67, 41.0/58.3, and 4.55/50.9 for the tumor model, respectively, and 1.87/9.54, 2.48/21.7, and 7.49/22.3 for the gray matter model, respectively.

### Uniqueness of the IVIM Parameters

**Figure 4:**
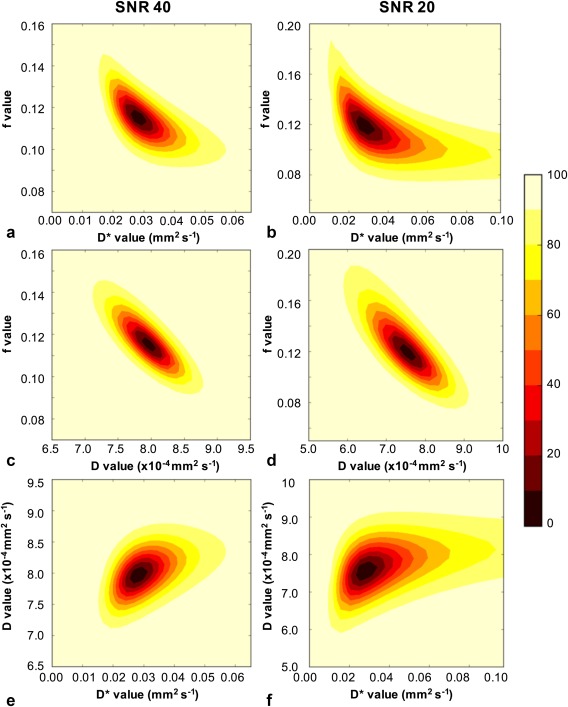
An example of one‐parameter fitting and the resulting error norm plots for gray matter model at SNR 40 (**a,c,e**) and SNR 20 (**b,d,f**) where data fitting was starting to fail due to greater amount of random noise. The plots were computed for all three IVIM parameter combinations of *f*‐D* (a,b), *f*‐*D* (c,d), and *D*‐D* (e,f). The contour colors describe the percentage confidence as shown by the color bar.

Confidence intervals were computed for the IVIM parameters at different SNR levels to investigate their uniqueness. An example case of 2D‐confidence interval or error‐norm plots with the gray matter model (based on one‐parameter fitting) is shown in Figure 4 for SNR levels of 40 (a–c) and 20 (d–f). The plots represent signal data, where data fitting was starting to fail due to a greater amount of random noise (“worst case scenario”). The most robust parameters were the IVIM‐*D* and IVIM‐*f*, which varied little within the 95.0% confidence region (Fig. 4c,d). However, the IVIM‐D* was increasingly overestimated at decreasing IVIM‐*f* (Fig. 4b) or increasing IVIM‐*D* (Fig. 4f) values, which corresponded to decreasing bi‐exponential behavior. Similar behavior was observed across all three bi‐exponential fitting methods. The error‐norm plots for the tumor model can be found in the Supplementary Material, which is available online. The plots showed multiple minima in the fitting of the IVIM parameters, which resulted in a wider distribution of estimated values, also observed as an increased number of outliers (Table 1).

**Figure 5:**
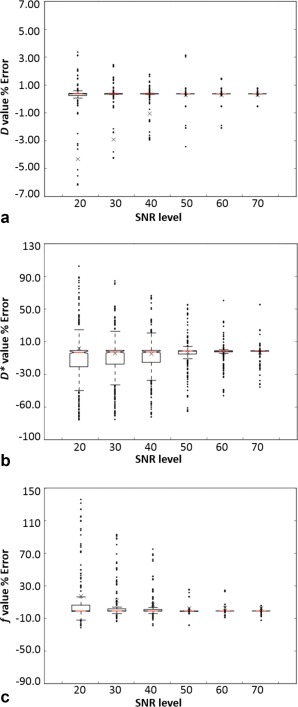
Boxplots for IVIM‐*D* (**a**), IVIM‐D* (**b**), and IVIM‐*f* (**c**) derived using the one‐parameter fitting with the gray matter model at increasing SNR levels, with the *y‐*axis describing the error to the true value. The crosses are the means, the central lines (red) are the medians, and the notches describe the 95% confidence levels in the median. The box edges are the first (Q1) and the third (Q3) data quartiles, with whiskers showing the more extreme data points not considered outliers.

The distributions of the estimated IVIM parameters at increasing SNR levels are shown in Figure 5 for the one‐parameter fitting. The estimation of the IVIM‐*D* values (Fig. 5a) was robust with few outliers, while a wider spread of values was observed for both IVIM‐D* (Fig. 5b) and IVIM‐*f* (Fig. 5c). The IVIM‐*f* value distributions were found positively skewed at the reducing SNR levels (opposite to the negatively skewed IVIM‐*D*) and the median gave a better measure of the central tendency. This was similarly observed in Figure 4 as the elongation of the confidence levels toward greater IVIM‐*f* values. With IVIM‐*D* and IVIM‐*f* fixed in the one‐parameter fitting, the estimation of IVIM‐D* is dependent on both. Therefore, not surprisingly a larger number of outliers was observed for the IVIM‐D*. However, the IVIM‐D* had limited robustness even at the higher SNR levels.

### Patient Imaging

**Figure 6:**
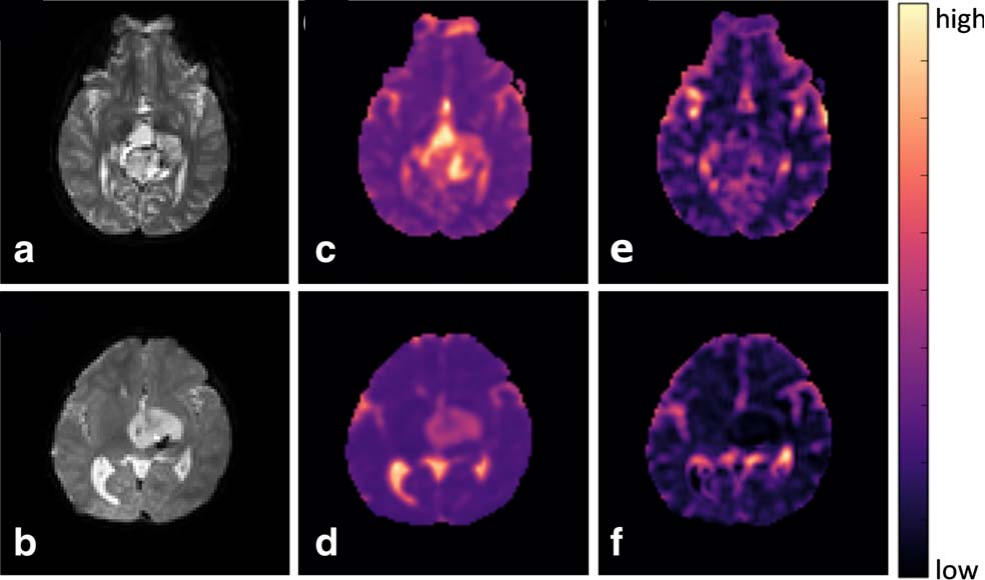
Example cases of glioneuronal tumor (top) and hypothalamic glioma (bottom) shown on *b*_0_ images (**a,b**), IVIM‐*D* maps (**c,d**), and IVIM‐*f* maps (**e,f**).

Two example cases of pediatric patients with assumed glioneuronal tumor and low‐grade hypothalamic glioma are shown in Figure 6. The maps were computed with the one‐parameter fitting. The IVIM‐*D* maps (Fig. 6c,d) show regions of low and high tissue diffusivity, which could be representative of the high and low cellularity within the tumors. The IVIM‐*f* maps (Fig. 6e,f) show areas of hypo‐perfusion, in particular for the glioma case.

## Discussion

Fitting of multi *b*‐value DWI for the determination of IVIM parameters was undertaken to evaluate the robustness of the fitting methods in low‐perfused tissue. The simulations showed that the constrained one‐ and two‐parameter fitting methods performed similarly for the tumor and gray matter model data. However, the estimated parameters from these methods differed significantly to the three‐parameter fitting derived parameters. The use of constrained fitting methods reduced the number of outliers, increased accuracy and provided more reproducible results in the estimation of IVIM parameters. Therefore, the more constrained methods provided more robust results compared with the simultaneous fitting.

The IVIM studies of abdomen and body^14^, ^24^, ^25^, ^26^ have often used the one‐parameter fitting to increase the reliability of the IVIM‐D* parameter at higher levels of perfusion. However, the reproducibility of IVIM‐D* in the gray matter model was found to be similar across the three fitting methods. Contrary to prior studies, the constrained fitting methods and in particular the one‐parameter fitting were found to produce more reliable results for the tumor model with the subtle bi‐exponential signal. This was observed with the estimation of the IVIM‐D* and to a lesser extent with IVIM‐*f*. Therefore, the subtle bi‐exponential signal observed in tissues of low perfusion can benefit from the use of more constrained fitting methods.

Results from the data simulations showed that the estimation of IVIM‐*D* and IVIM‐*f* was more robust and reliable compared with IVIM‐D*. Both IVIM‐*f* and IVIM‐D* were affected by the degree of perfusion or bi‐exponential behavior, which resulted in greater variance for the low‐perfused tumor model. However, the IVIM‐*f* was still estimated with good accuracy at a lower SNR using the one‐parameter fitting method. A similar perfusion‐related influence was observed in a study by Wu et al,^11^ where simulations showed greater variance for the less‐perfused white matter compared with gray matter. Therefore, low‐perfused tissues have a higher SNR requirement for the computation of reliable IVIM derived parameters. Overall, our simulations suggest that the one‐parameter fitting method can provide the most reliable results with the smallest number of outliers, followed by the two‐parameter fitting, which has been more commonly applied in the brain.^12^, ^13^

The uniqueness of the IVIM parameters was investigated using error norm plots. For models such as bi‐exponential, the approximation of the standard error from the covariance matrix can begin to fail and confidence intervals can provide a better measure of robustness of the parameters. Most of the gray matter and tumor model iterations produced symmetrical uncertainties and the standard errors derived from the covariance matrix produced sufficient approximates of uncertainties. Therefore, the presented gray matter and tumor model cases do not reflect the majority of the data observed at the corresponding SNR levels, but rather indicated the behavior of the models at high random noise levels.

In the case of the tumor model, multiple minima were observed in the estimation of the IVIM parameters. The convergence of the algorithm to these local minima at lower SNR levels was likely to affect the overall mean values, as the distribution of the estimated IVIM parameters was shifted. The application of multi‐compartmental models to MRI data have been previously studied by Jelescu et al,^27^ who showed that the global minima was not always the correct solution. However, the convergence to the correct minimum is important for the clinical reliability of the model. In our tumor simulations, there was only a single minimum for cases with SNR > 50 and at SNR 40 multiple minima were observed for < 7% of cases. In the cases of multiple minima, the global minimum was also found to be correct. Therefore, an experimental solution would be to improve the SNR by increasing the number of averages of the acquired signal data. This would minimize the number of multiple minima cases, improve the data fitting and increase the reliability of the model as shown in the simulations.

The behavior of the gray matter model showed that the estimation of IVIM‐*D* and IVIM‐*f* was still robust in the presence of noise. A loss in uniqueness of the IVIM‐D* was observed, with a range of IVIM‐D* values estimated for fixed IVIM‐*f* and IVIM‐*D* values. This was also illustrated as the large number of outliers for IVIM‐D*. In comparison to the other fitting methods, the constrained one‐parameter fitting provided the best estimate of IVIM‐D*. With the limited robustness and loss in uniqueness of IVIM‐D*, we concluded it did not provide a reliable measure of microvascular diffusion for the low perfusion regime in the brain. An increase in the number of low *b*‐values could potentially increase the robustness of IVIM‐D*, which was previously shown in the liver study by Cohen et al.^14^ Also, as previously mentioned, an increase in the number of averages would improve the fitting and, hence, the robustness of the IVIM‐D* and IVIM‐*f*. An alternative approach would be to use the IVIM model as a complimentary technique to arterial spin labeling (ASL‐MRI). This would provide a more complete noninvasive investigation of perfusion with the computation of IVIM‐*f* (comparable to CBV) and ASL‐CBF parameters.

The limitations in the fitting methods used in this study include the use of a model that assumes the signal is bi‐exponential. In regions where the signal decay was or approaches mono‐exponential behavior, the accuracy, and reproducibility of the estimated IVIM‐*f* and IVIM‐D* values was decreased. The IVIM‐*f* was affected to a lesser degree than the IVIM‐D*, which performed poorly in the model simulations. Another issue encountered in the brain was the presence of cerebrospinal fluid (CSF). In most cases, this caused the fitting to estimate values of IVIM‐*f* that were higher than 0.3 and a threshold was introduced to remove this “nonphysiological” data. However, partial volume effects from CSF were found to be problematic in some areas. A more sophisticated approach, which could determine the number of exponential components from the data might improve the fitting of the low‐perfused regions and potentially provide more reliable results. The IVIM model used in this study did not take into account any relaxation time effects, which were previously found to affect the perfusion‐related parameters in the abdominal organs.^28^ This was based on the more recent findings by Orton et al,^29^ who showed statistical support for the extended IVIM model in the liver, but not for the lower perfused organs (spleen and kidney). Therefore, these effects are likely to be negligible in the low‐perfused tissues investigated in this study.

In conclusion, based on the simulated data, the best performing fitting algorithm was the constrained one‐parameter method, which resulted in the most reliable IVIM parameters. The estimated IVIM parameters had similar accuracy (mean error and bias) between the fitting methods, but differences were observed in the reproducibility and number of outliers. While the lower degree of bi‐exponential behavior with the tumor model resulted in a larger variance in the IVIM parameters, the estimation of IVIM‐*D* and IVIM‐*f* was robust. The data simulations and also the presence of local minima indicated that there is an SNR requirement for reliable IVIM data analysis (SNR > 40). This study showed that the constrained fitting of the IVIM model can be used to assess low‐perfused tissues.

## Supporting information

Additional supporting information may be found in the online version of this article

Supporting Information

Supporting Information Figure S1
