# Supplementary material for: Evaluation of intravoxel incoherent motion fitting methods in low‐perfused tissue
Source: J Magn Reson Imaging. 2016 Aug 22;45(5):1325–34. doi: 10.1002/jmri.25411 (PMC5412931; doi:10.1002/jmri.25411)
Supplement: Supplementary file 1 — Supporting Information [file JMRI-45-1325-s001.docx]

Supplementary Figure. An example of 1-parameter fitting and the resulting error norm plots for tumour model at SNR 40 (a,c,e) and SNR 20 (b,d,f) where data fitting was starting to fail due to greater amount of random noise. The plots were computed for all three IVIM parameter combinations of *f*-*D** (a,b), *f*-*D* (c,d) and *D­-D** (e,f). The contour colours describe the percentage confidence as shown by the colour bar.
